# Predicting enhancers with deep convolutional neural networks

**DOI:** 10.1186/s12859-017-1878-3

**Published:** 2017-12-01

**Authors:** Xu Min, Wanwen Zeng, Shengquan Chen, Ning Chen, Ting Chen, Rui Jiang

**Affiliations:** 1 MOE Key Laboratory of Bioinformatics and Bioinformatics Division, TNLIST, Beijing, 100084 China; 2 Department of Computer Science and Technology, State Key Lab of Intelligent Technology and Systems, Tsinghua University, Beijing, 100084 China; 3 Department of Automation, Tsinghua University, Beijing, 100084 China; 4 Program in Computational Biology and Bioinformatics, University of Southern California, Los Angeles, CA 90089 USA

## Abstract

**Background:**

With the rapid development of deep sequencing techniques in the recent years, enhancers have been systematically identified in such projects as FANTOM and ENCODE, forming genome-wide landscapes in a series of human cell lines. Nevertheless, experimental approaches are still costly and time consuming for large scale identification of enhancers across a variety of tissues under different disease status, making computational identification of enhancers indispensable.

**Results:**

To facilitate the identification of enhancers, we propose a computational framework, named DeepEnhancer, to distinguish enhancers from background genomic sequences. Our method purely relies on DNA sequences to predict enhancers in an end-to-end manner by using a deep convolutional neural network (CNN). We train our deep learning model on permissive enhancers and then adopt a transfer learning strategy to fine-tune the model on enhancers specific to a cell line. Results demonstrate the effectiveness and efficiency of our method in the classification of enhancers against random sequences, exhibiting advantages of deep learning over traditional sequence-based classifiers. We then construct a variety of neural networks with different architectures and show the usefulness of such techniques as max-pooling and batch normalization in our method. To gain the interpretability of our approach, we further visualize convolutional kernels as sequence logos and successfully identify similar motifs in the JASPAR database.

**Conclusions:**

DeepEnhancer enables the identification of novel enhancers using only DNA sequences via a highly accurate deep learning model. The proposed computational framework can also be applied to similar problems, thereby prompting the use of machine learning methods in life sciences.

## Background

Enhancers are short DNA sequences that can be bound by transcription factors to boost the expression of their target genes. Recent advances in the study of gene regulatory mechanisms have suggested that enhancers are typically 50-1500 bp long, located either upstream or downstream from the transcription start site of their target genes. Besides, enhancers are believed to cooperate with promoters to regulate the transcription of genes in a cis-acting and tissue specific manner, making these short sequences crucial in the understanding of gene regulatory mechanisms, and thus receiving more and more attentions in not only genomic and epigenomic studies but also the deciphering of genetic basis of human inherited diseases [1–3].

The identification of enhancers is usually done by using high-throughput sequencing techniques. For example, Heintzman and Ren used ChIP-seq experiments to establish a landscape of binding sites for individual transcription factor [4]. However, it is not practical to identify all enhancers using this approach because the knowledge of a subset of transcription factors that occupy active enhancer regions in a specific cell line must be known *a prior*. May et al. mapped the binding sites of transcriptional coactivators such as EP300 and CBP that are recruited by sequence-specific transcription factors to a large number of enhancers [5]. Nevertheless, it is known that not all enhancers are marked by a given set of co-activators, and thus systematic identification of enhancers using this approach is not feasible. Recent advances in epigenomics also suggest the approach of identifying enhancers relying on chromatin accessibility, usually resorting to such innovative techniques as DNase-seq [6]. However, this approach is not specific to enhancers because accessible chromatin regions may also correspond to promoters, silencers, repressors, insulators, and other functional elements. With the recognition that active promoters are marked by trimethylation of Lys4 of histone H3 (i.e., H3K4me3), whereas enhancers are marked by monomethylation instead of trimethylation of H3K4 (i.e., H3K4me1) [7], genome-wide identification of enhancers have been conducted in large-scale projects such as ENCODE (Encyclopedia of DNA Elements) and Roadmap [8]. Besides, using an experimental technique called cap analysis of gene expression (CAGE), the FANTOM project has successfully mapped promoters and enhancers that are active in a majority of mammalian primary cell lines [9].

However, experimental approaches are expensive and time consuming for large scale identification of active enhancers across a variety of human tissues and cell lines. In spite of great efforts, the ENCODE and Roadmap projects were only able to carry out histone modification experiments in several hundred human cell lines thus far, still far less than forming a comprehensive landscape of enhancers under different disease status and subsequently preventing the deciphering of gene regulatory mechanisms. To address this problem, computational approaches have been proposed to conduct *in silicon* prediction of enhancers by using DNA sequences. To mention a few, Lee et al. developed a computational framework called kmer-SVM based on the support vector machine (SVM) to discriminate mammalian enhancers from background sequences [10]. They found that some predictive k-mer features are enriched in enhancers and have potential biological meaning. Ghandi et al. improved kmer-SVM by adopting another type of sequence features called gapped k-mers [11]. Their method, known as gkmSVM, showed robustness in the estimation of k-mer frequencies and allowed higher performance than kmer-SVM. However, k-mer features, though unbiased, may lack the ability to capture high order characteristics of enhancer sequences.

With the rapid development of deep learning since early 2000s, many researchers have tried to apply the state-of-the-art deep learning method in bioinformatics problems. For example, Quang et al. annotated the effect of noncoding genetic variants by training a deep neural network [12]. Their method achieved higher performance than the traditional machine learning method CADD [13]. In DeepBind [14], Alipanahi et al. used a deep learning strategy to predict DNA- and RNA-binding proteins from diverse experimental data sets. The results showed that deep learning methods have broad applicability and improved prediction power than traditional classification methods. Besides, Zhou et al. developed a deep-learning method, named DeepSEA, that learned a regulatory sequence code from large-scale chromatin-profiling data including histone modification, TF binding, etc. to predict effects of noncoding variants [15]. For example, Kelley *el al.* proposed a method called Basset that applies deep convolutional neural networks to learn functional activities of DNA sequences from genomics data [16]. All these methods suggest that deep learning provides a powerful way to carry out genomics studies, stimulating us to ask the question of whether enhancers can be identified merely by sequence information.

Motivated by the above understanding, in this paper, we propose a method called DeepEnhancer to predict enhancers using a deep convolutional neural network (CNN) framework. Specifically, we regard a DNA sequence as a special 1-D image with four channels corresponding to four types of nucleotides and train a neural network model to automatically distinguish enhancers from background genome sequences in different cell lines. Unlike a traditional classifier such as the support vector machine, our method skips the handcrafted feature extraction step. Instead, we use convolutional kernels to scan input short DNA sequence and automatically obtain low level motif features, which are then fed to a max pooling layer and eventually to densely connected neurons to generate high level complex features through a nonlinear activation function. To gain interpretability of our method, we design a visualize strategy that extracts sequence motifs form kernels in the first convolutional layer. We evaluate the performance of our method using a large set of permissive enhancers defined in the FANTOM5 project [9]. Results, quantified by such criteria as the area under the receiver operation characteristic curve (AUROC) and that under the precession recall curve (AUPRC), strongly support the superiority of our method over traditional classifiers. Taking tissue specificity of enhancers into consideration, we adopt a transfer learning strategy to fine-tune our model for 9 datasets of enhancers specific to a variety of cell lines in the ENCODE project [17]. Corresponding results also support the high performance of our method. We expect to see wide applications of our approach to not only genomic and epigenomic studies for deciphering gene regulation code, but also human and medical genetics for understanding functional implications of genetic variants.

## Results

### Overview of DeepEnhancer

**Fig. 1:**
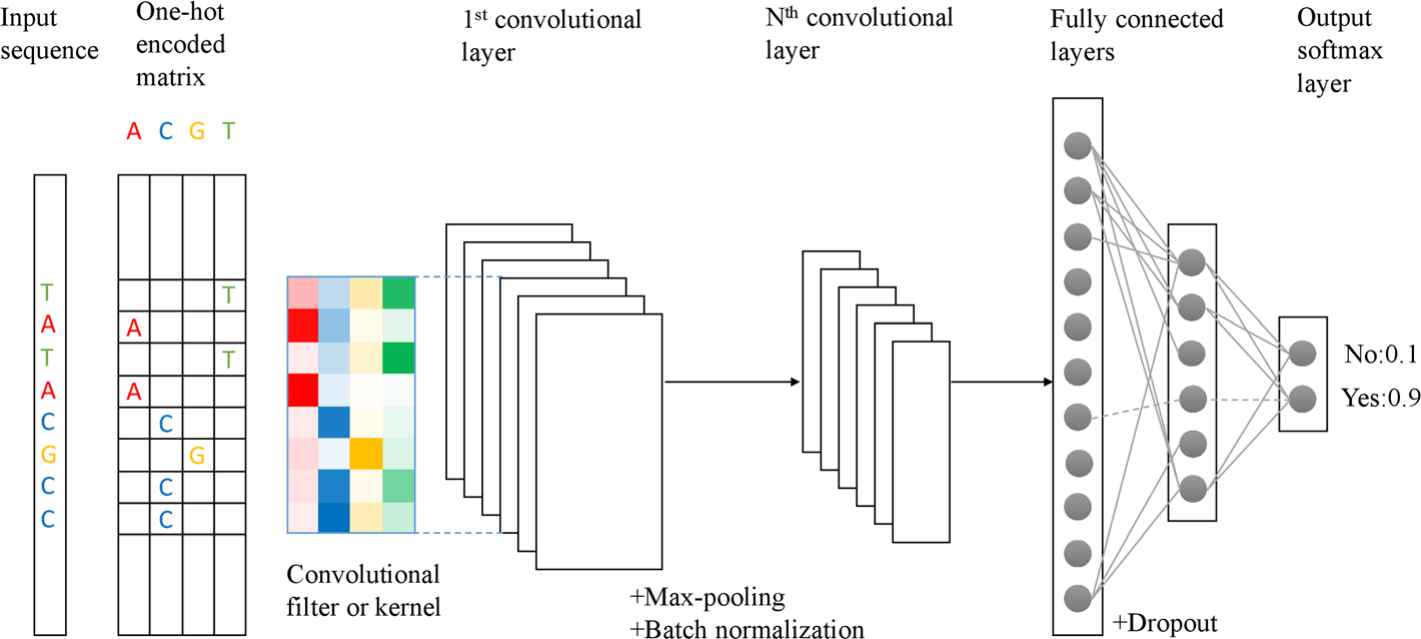
Overview of DeepEnhancer. A raw DNA sequence is first encoded into a binary matrix. Kernels of the first convolutional layer scan for motifs on the input matrix by the convolution operation. Subsequent Max-pooling layer and batch normalization layer are used for dimension reduction and convergence acceleration. Additional convolutional layers will model the interaction between motifs in previous layers and obtain high-level features. Fully-connected layers with dropout will perform nonlinear transformations and finally predict the response variable through softmax layer

As illustrated in Fig. 1, DeepEnhancer, the proposed deep convolutional neural network model, is composed of multiple convolutional layers, max-pooling layers, and fully connected layers. In the first convolutional layer, a number of convolutional kernels or filters are used to scan along an input sequence for short sequence patterns. In each of the subsequent convolutional layers, low level patterns from the previous layer are further scanned to capture high level patterns. In each layer, a batch normalization operation is performed to restrict output values not exceeding the maximum. In a max-pooling layer, input patterns are reduced to a low dimension, for the purpose of alleviating computational burden and facilitating the extraction of high level features. In a fully connected layer, input variables are discarded at random by a dropout operation, fed to a rectified linear unit (ReLU) for incorporating nonlinear flavor, and eventually transformed into probabilities through a softmax function.

A hallmark of our model is the use of convolutional kernels. Opposed to traditional classification approaches that are based on elaborately-designed and manually-crafted features, convolutional kernels perform adaptive learning for features, analogous to a process of mapping raw input data to informative representation of the knowledge. In this sense, the convolutional kernels can be thought of as a series of motif scanners, since a set of such kernels is capable of recognizing relevant patterns in the input and updating themselves during the training procedure.

A deep convolutional neural network typically has a vast number of parameters. As described in Table 1, in our model, the input layer is a 4 × 1 × *L* matrix, where *L*, with the default value of 300, is the length of the input sequence. The four types of nucleotides, A, C, G, and T, are encoded by using the one hot method, forming 4 channels. Therefore, a short sequence of length *L* can be thought of as an image of 4 channels with height 1 and width *L*. The first convolutional layer contains 128 kernels of shape 1 × 8, with sliding step 1. Right behind the first convolutional layer is a batch-normalization layer, which is followed by another convolutional layer with 128 kernels of shape 1 × 8. After a max-pooling layer with pooling size 1 × 2, there are two other convolutional layers with 64 kernels of shape 1 × 3. Like the first convolutional layer, each of the four convolutional layers is followed by a batch-normalization layer. On the top of the architecture are two fully connected layers of size 256 and 128, respectively, with a dropout layer (ratio 0.5) between them. The final 2-way softmax layer generates the classification probability results.

**Table 1:** Different network architectures of DeepEnhancer

Layer ID	Layer Type	Size	Output shape
0	Input	–	4x1x300
1	Conv	128x4x1x8	128x1x293
2	Batchnorm	–	128x1x293
3	Conv	128x128x1x8	128x1x286
4	Batchnorm	–	128x1x286
5	Maxpooling	1 × 2	128x1x143
6	Conv	64x128x1x3	64x1x141
7	Batchnorm	–	64x1x141
8	Conv	64x64x1x3	64x1x139
9	Batchnorm	–	64x1x139
10	Maxpooling	1 × 2	64x1x69
11	Dense	256	256
12	Dropout	–	256
13	Dense	128	128
14	Softmax	2	2

The size column records the convolutional kernel size, the max-pooling window size and the fully connected layer size. The output shape depicts the change of data’s shape in the flow

### DeepEnhancer predicts permissive enhancers

We evaluated our method using a set of 43,011 permissive enhancers obtained from the FANTOM5 project. For this objective, we labelled sequences of these enhancers as positive and sampled from the human reference genome (GRCh37/hg19) the same number of sequences as negative, obtaining a dataset for evaluation. We then carried out a 10-fold cross-validation experiment for each architecture of the neural network using the evaluation data. Briefly, we partitioned the dataset into 10 subsets of nearly equal size. In each fold of the experiment, we took 9 subsets to train the CNN model and tested its performance using the remaining subset. Particularly, in the training phase, we first converted training sequences of variable length to short sequences of fixed length using a pipeline detailed in the data processing section and then fed the resulting data to the CNN. In the test phase, we also converted a test region to multiple short sequences and then assigned the maximum prediction probability of such short sequences to the test region.

We implemented DeepEnhancer by using a well-known wrapper called Lasagne [18], which is built on top of Theano [19, 20]. In the training phase, we resorted to the recently proposed Adam algorithm [21] for the stochastic optimization of the objective loss function, with the initial learning rate setting to 10^−4^ and the max number of epochs setting to 30. We also applied the learning rate decay schedule and the early stopping strategy to accelerate the convergence of training.

**Fig. 2:**
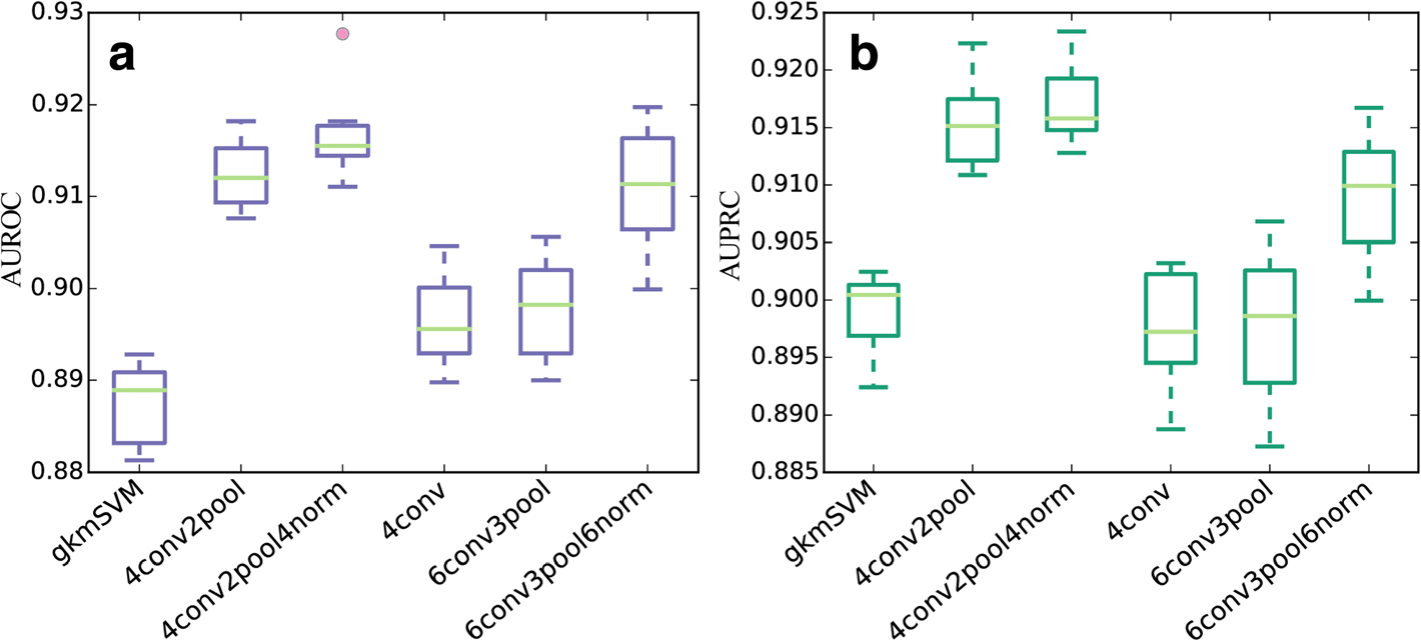
AUROCs of different methods on the permissive enhancer dataset. **a**: boxplot for AUROC scores. **b**: boxplot for AUPRC scores. The main body of the boxplot shows the quartiles. The horizontal lines at the median of each box show the medians. The vertical lines extending to the most extreme represent non-outlier data points

We compared the performance of 5 network architectures described in the methods section and the gapped k-mer support vector machine (gkmSVM) [11], which were regarded as the state-of-the-art sequence-based model for predicting regulatory elements. In the comparison, the performance of a method was evaluated in terms of two criteria, AUROC (the area under the receiver operating characteristic curve) and AUPRC (the area under the precision-recall curve). As shown in Table 2 and Fig. 2, we found that our deep learning models of different architecture all surpassed the conventional sequence-based method of gkmSVM. Specifically, the model 4conv2pool4norm achieved the highest performance with a mean AUROC of 0.916 and a mean AUPRC of 0.917. Even the model with the lowest performance, 4conv, yielded a slightly higher performance than gkmSVM. We then carried out pairwise Wilcoxon tests on the AUROC and AUPRC scores of gkmSVM and the five CNN models. As shown in Tables 3 and 4, pairwise Wilcox rank-sum tests also suggest that the model 4conv2pool4norm outperforms the gkmSVM baseline, and the results are statistically significance, suggest the superiority of the deep learning method over traditionally binary classification approach. Besides, DeepEnhancer, as a typical deep learning method, does not require any pre-defined features such as k-mer counts used by gkmSVM. With convolution kernels, our method can adaptively learn high-quality features from the large-scale dataset and then use them for accurate classification.

**Table 2:** Classification performance for different network architectures

Model	AUROC	AUPRC	Epoch Time
gkmSVM	0.887 (0.004)	0.899 (0.004)	6 h (total)
4conv2pool	0.910 (0.004)	0.915 (0.004)	272 s
4conv2pool4norm	0.916 (0.004)	0.917 (0.003)	376 s
4conv	0.896 (0.005)	0.897 (0.005)	325 s
6conv3pool	0.898 (0.005)	0.898 (0.006)	251 s
6conv3pool6norm	0.911 (0.006)	0.909 (0.005)	415 s

The conventional gkmSVM is used as the baseline for comparison. For each model, we carried out 10-fold cross validation experiments. This table records the mean value of AUC values with standard error behind in the brackets

**Table 3:** Pairwise Wilcoxon tests on AUROCs of different methods

	gkmSVM	4conv2pool	4conv2pool4norm	4conv	6conv3pool	6conv3pool6norm
gkmSVM	–	5.1e-3	5.1e-3	5.1e-3	5.1e-3	5.1e-3
4conv2pool	–	–	4.6e-2	5.1e-3	5.1e-3	9.6e-1
4conv2pool4norm	–	–	–	5.1e-3	5.1e-3	2.8e-2
4conv	–	–	–	–	2.4e-1	5.1e-3
6conv3pool	–	–	–	–	–	6.9e-3
6conv3pool6norm	–	–	–	–	–	–

We perform pairwise Wilcoxon tests on AUROCs of the six methods. Tests are conducted with the alternative hypothesis that the AUROCs of two methods are different in their medians. Small *p*-values indicate that two methods have different performance

**Table 4:** Pairwise Wilcoxon tests on AUPRCs of different methods

	gkmSVM	4conv2pool	4conv2pool4norm	4conv	6conv3pool	6conv3pool6norm
gkmSVM	–	5.1e-3	5.1e-3	6.5e-1	5.8e-1	5.1e-3
4conv2pool	–	–	2.8e-1	5.1e-3	5.1e-3	5.1e-3
4conv2pool4norm	–	–	–	5.1e-3	5.1e-3	5.1e-2
4conv	–	–	–	–	4.4e-1	5.1e-3
6conv3pool	–	–	–	–	–	9.3e-3
6conv3pool6norm	–	–	–	–	–	–

We perform pairwise Wilcoxon tests on AUPRCs of the six methods. Tests are conducted with the alternative hypothesis that the AUPRCs of two methods are different in their medians. Small *p*-values indicate that two methods have different performance

Moreover, the comparison between different architectures of the neural network suggested that the pooling operation increases the classification performance, since the model 4conv without pooling layers was obviously inferior to model 4conv2pool. The pooling operation helps to abstract features in the previous layer and increases the receptive field, hence it improves representation power of our method. In addition, we also noted that the batch normalization strategy used in 4conv2pool4norm and 6conv3pool6norm did improve the performance of a model. Surprisingly, while deeper models usually achieved better performance, we observed that a model with 6 convolution layers (6conv3pool) demonstrated inferior performance when compared with a model with 4 convolutional layers (4conv2pool). Similarly, we observed that the model 6conv3pool6norm achieved lower performance than 4conv2pool4norm. We conjectured that more training data may be necessary in order to train an even deeper architecture.

### DeepEnhancer predicts cell line specific enhancers

It is well known that a hallmark of enhancers is the tissue specificity. Although our model has successfully exhibited the power of distinguishing permissive enhancers from background random sequences in the above section, whether enhancers specific to a tissue or cell line can also be identified using our model remains a question. Directly applying the deep learning model to enhancers specific to a tissue may not succeed, because the number of enhancers known to be specific to a tissue is in general quite limited, and thus greatly restricts the complexity of the model. We therefore adopted a transfer learning strategy to borrow models well-trained in permissive enhancers, for the purpose of reducing the model complexity. This idea is analogous to a lot of successful studies in computer vision, where very few people train an entire convolutional neural network from scratch with random parameter initialization, since it is relatively rare to get a dataset of sufficient size. Instead, it is common to use a CNN model pre-trained on a very large dataset, such as ImageNet, which contains about 1.2 million images and 1000 categories [22].

With the transfer learning strategy, we first trained a model (4conv2pool4norm) using the dataset of permissive enhancers and then fine-tuned the weights of the resulting model by continuing the back propagation on a dataset of enhancers specific to a certain cell line. Note that permissive enhancers in FANTOM5 are all experimentally verified, while enhancers specific to a cell line are predicted by the ChromHMM model, which may have lower accuracy. However, by fine-tuning, we can fuse the trustable knowledge we distilled from permissive dataset into the training of the cell line specific models.

**Fig. 3:**
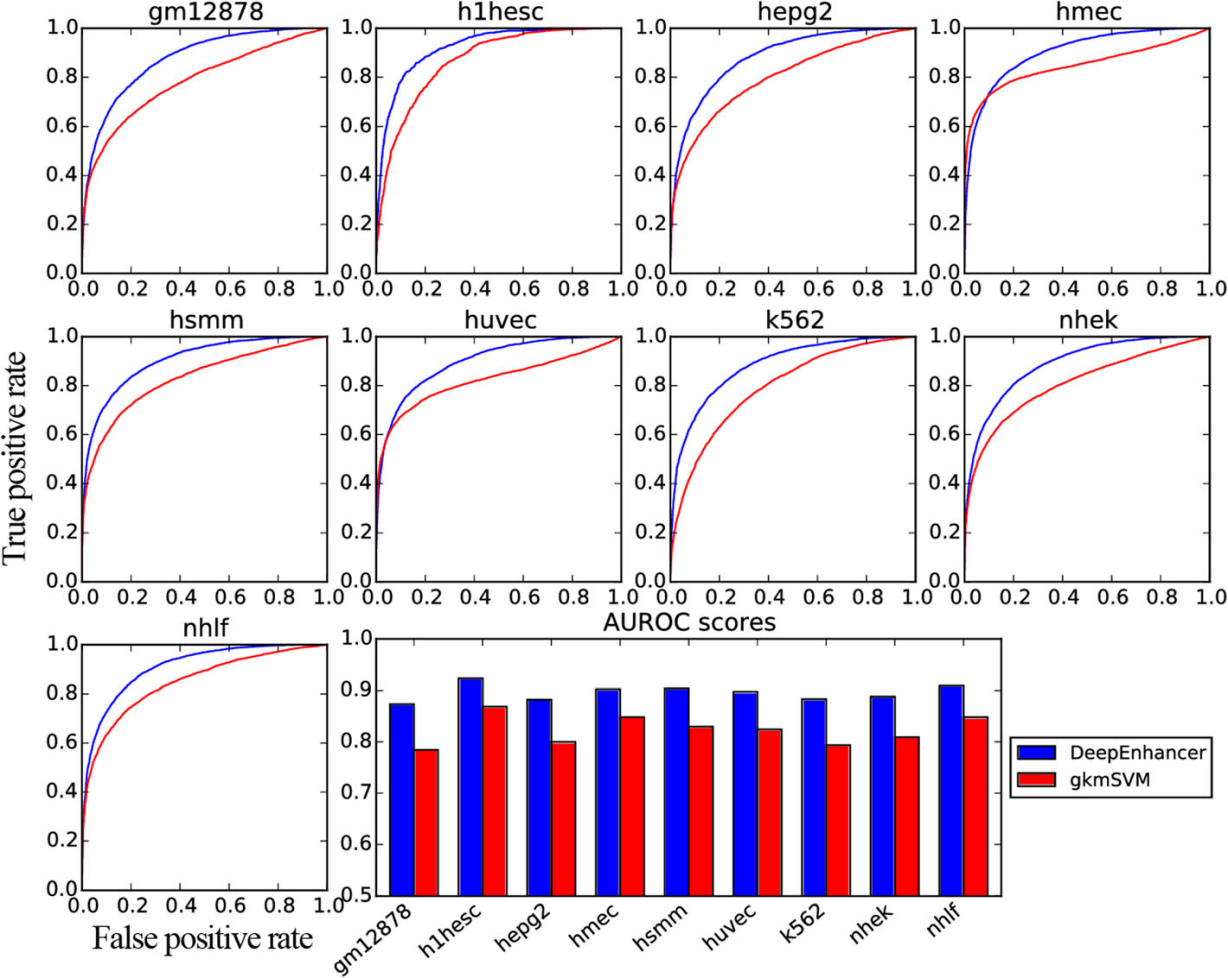
ROC curves for enhancers specific to different cell lines. The first nine subplots depict the receiver operating characteristic (ROC) curves, and the last subplot is the barplot of the AUROC

**Fig. 4:**
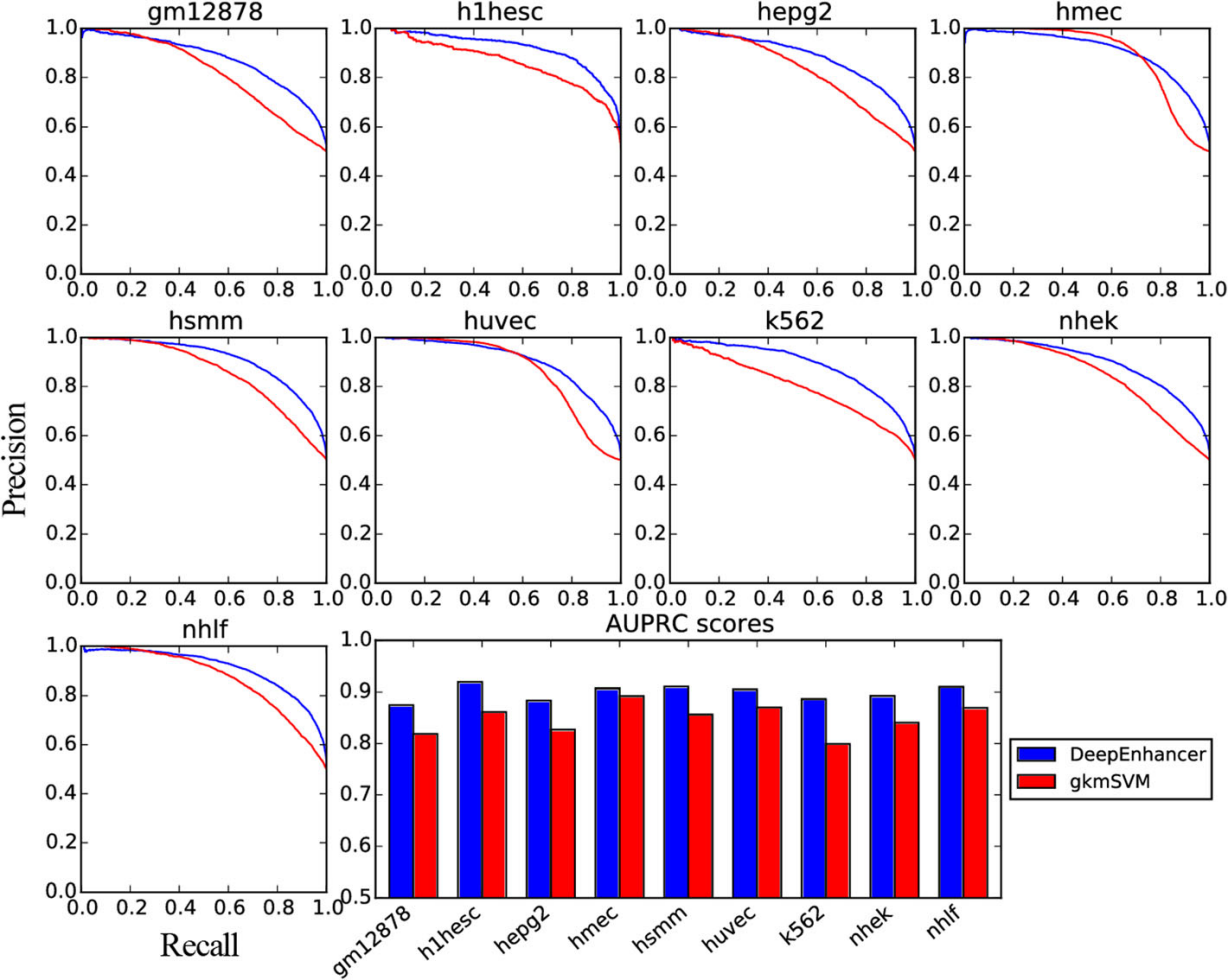
PR curves for enhancers specific to different cell lines. The first nine subplots depict the precision-recall (PR) curves for the 9 cell types respectively, and the last subplot is the barplot for AUPRC

As shown in Table 5, the fine-tuned CNN models unexpectedly achieves higher performance than gkmSVM for enhancers specific to 9 different cell lines, say, GM12878, H1-hESC, HepG2, HMEC, HSMM, HUVEC, K562, NHEK, and NHLF. Taking GM12878 as an example, our model achieves an AUROC of 0.874 and an AUPRC of 0.875, while gkmSVM only achieves an AUROC of 0.784 and an AUPRC of 0.819. On average, our method is superior to gkmSVM by about 7% in both AUROC and AUPRC scores. We then counted the number of cell lines that our method achieved a higher AUROC than gkmSVM and conducted a Binomial exact test against the alternative hypothesis that the probability that our model outperformed gkmSVM is greater than 0.5. The small *p*-value (1.9×10^−3^) supports the significance of the test and suggests the superiority of our method over gkmSVM. A similar test regarding AUPRC gave us a similar conclusion. Furthermore, receiver operating characteristic curves for the 9 cell lines, as depicted in Fig. 3, clearly show that our method produces curves that climb much faster towards to top-left corner of the sub-plots, suggesting that our method can achieve relatively high true positive rate at relatively low false positive rate. Precision-recall curves for individual cell lines, as shown in Fig. 4, also suggest the superiority of our method. From these results, we concluded that our deep learning model is more powerful in modeling genomic sequences than conventional k-mer based methods.

**Table 5:** Classification performance for different cell lines

Cell Type	AUROC		AUPRC	
	DeepEnhancer	gkmSVM	DeepEnhancer	gkmSVM
GM12878	0.874	0.784	0.875	0.819
H1-hESC	0.923	0.869	0.919	0.861
HepG2	0.882	0.800	0.883	0.827
HMEC	0.903	0.848	0.907	0.892
HSMM	0.904	0.830	0.910	0.856
HUVEC	0.898	0.824	0.905	0.870
K562	0.883	0.794	0.886	0.799
NHEK	0.888	0.809	0.893	0.840
NHLF	0.909	0.848	0.910	0.869
*p*-value	1.9e-3		1.9e-3	

We compare the performance of our DeepEnhancer model and gkmSVM on 9 cell types using two measures: area under receiver operating characteristic curve (AUROC) and area under precision-recall curve (AUPRC). The last row shows the p-value result of the binomial exact test, which makes us choose the alternative hypothesis that DeepEnhancer has a larger AUC score than gkmSVM

### DeepEnhancer learns sequence motifs

A debate regarding deep learning methods is the weak interpretability, that is, features used by dense layers of a convolutional neural network may hard to understand. To gain the interpretability of our models in the above two sections, we proposed a strategy to visualize sequence motifs recovered by our model as sequence logos. Briefly, inspired by related studies in computer vision [23, 24], Lanchatin et al. addressed the sequence visualization problem by solving an optimization problem that found the input matrix corresponding to the highest probability of transcription factor binding sites via back propagation [25]. However, since we trained the network on binary matrix input, it seems a little weird to optimize the input matrix in a continuous space. We therefore proposed the following strategy to extract and visualize sequence motifs encoded in the first convolutional layer of our model.

Typically, a convolutional neural network model scans the input sequence **s** in a window with multiple convolutional kernels or filters with weights **W**, and then through an activation function, e.g., a rectified linear unit (ReLU), with bias **b** to obtain the output of the first layer, as

$$ \mathrm{Conv}1\left(\mathbf{s}\right)=\mathrm{ReLU}\left(\mathbf{s}\otimes \mathbf{W}+\mathbf{b}\right), $$

where symbol ⊗ means the convolution operation. Instead of searching for an input matrix in a continuous Euclidean space, we sought for all possible input matrices that have positive activation values through the first convolutional layer, and then aggregated them into a positive weight matrix (PWM) which is used to represent a motif. In detail, since our learned parameter **W** is in shape (128 × 4 × 1 × 8), it can be converted into 128 weight filters **w**i in shape (4 × 8). For each weight filter **w**i, we found all possible one-hot encoded input matrices s in shape (4 × 8) with positive convolutional activations, which represent motifs our model can identify. Note that our convolutional filter has width 8, the search space is limited to only 4,8 so traversal search operation can be fairly feasible. After we collected the PWMs for all the 128 weight filters, we evaluated our motifs by performing comparison against JASPAR motifs [26], which are widely known as the gold standard representations of positive binding sites for hundreds of transcription factors. In order to compute the similarity of our motifs, we used a tool called TOMTOM with predefined statistical measure of motif-motif similarity [27, 28]. TOMTOM compared a group of motifs in length 8 against motifs in JASPAR dataset whose lengths range in (5, 30) and produced an alignment for each significant match.

**Fig. 5:**
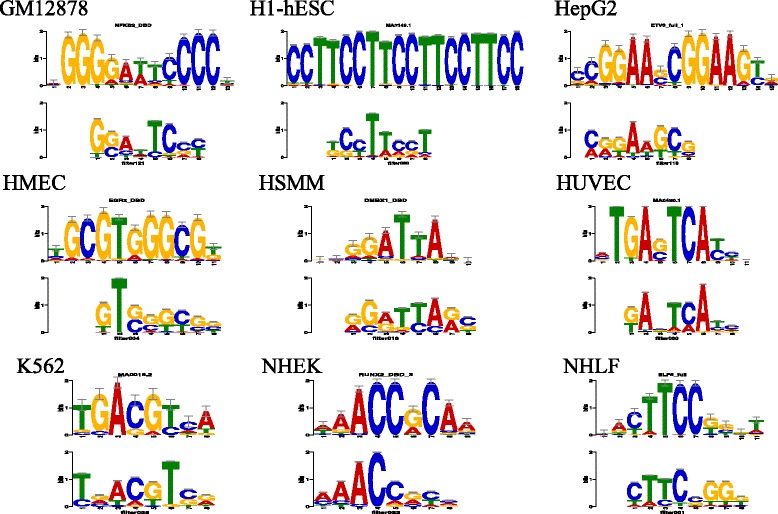
Visualization of learned motifs. For each cell line, we show a pattern learned by our model and can be matched to a known motif in the JASPAR database

**Fig. 6:**
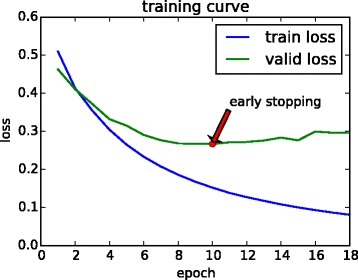
Loss of the model 4conv2pool4norm during training. The loss of the training set decreases rapidly, and we hold out a validation set for early stopping after 8 epochs of unimproved valid loss

In practice, for each cell line, we compared the motifs transformed by the first convolutional layer of our model against the Vertebrates (in vivo and in silico) motif database using TOMTOM, and set the significance threshold E-value <0.1. Results, as shown in Fig. 5, demonstrate that many of our learned motifs have significant similarity to the biologically known motifs. For example, the nuclear factor κB (NF-κB) has been detected in numerous cell types that express cytokines, chemokines, growth factors, cell adhesion molecules, and some acute phase proteins in health and in various disease states. In a recent study, Zhao et al. found that NF-κB are enriched at active enhancers, as characterized by H3K4me1 and H3K27ac marks by using validated GM12878 chromatin state annotations based on histone modifications [29], suggesting the prevalence of NF-κB motifs in enhancers specific to the GM12878 cell type. Interestingly, according to our visualization results (Fig. 6), we find the presence of a learned pattern which is very similar to the NF-κB motif in the GM12878 cell type, coinciding with the finding of Zhao et al. and revealing the power of our DeepEnhancer method in extracting sequence features. However, note that not all of learned motifs are precisely consistent with known motif databases. On one hand, the accuracy of learned motifs depends on the training dataset. On the other hand, our computational framework may uncover new motifs not experimentally verified yet.

### DeepEnhancer is efficient in computation time

It may be argued that the vast number of parameters in a deep convolutional neural network may greatly increase the computational burden. Nevertheless, in practical, with the use of high-performance NVIDIA Tesla K80 GPU, our DeepEnhancer model also gained superiority in the running-time over gkmSVM. Taking the 4conv2pool4norm model as an example, each training epoch costed about 376 s (Table 2), and we stopped model training at 18 epochs (Fig. 6) according to the early stopping strategy. Hence our model training totally consumed only less than 2 h. In contrast, gkmSVM took about 6 h on average until convergence. Hence, with the aid of computer hardware, our approach can allow researchers to train highly accurate deep models within quite a short time. Considering the vast amount of potential regulatory elements in the whole genome, this characteristic is particular useful when applying our model to study other types of regulatory elements.

## Discussion

The superiority of our method over traditional classification approach such as gkmSVM may be mainly attributed to the use of the deep convolutional neural network model, which discards the hand-crafted feature extraction strategy and is capable of exploring much more sequence properties that contributes to the final classification task. This end-to-end learning strategy, with the support of the vast amount of genomic big data and the rapid growing computing power, opens a door to large scale deciphering of sequence code and will eventually benefit a wide range of biological and medical studies [30–32]. Nevertheless, our study also emphasizes the importance of several techniques that are crucial to the success of a deep learning approach in genomic studies. For example, data augmentation seems indispensable, given the fact that the sample size in a biological experiment is typically small. Transfer learning, which can be thought of as a strategy for incorporating knowledge from closely related data, seems beneficial, especially when intrinsic properties of the data are consistent.

Our method has a wide range of applications in a variety of scenarios. First, our method can be used with such high-throughput sequencing techniques as ChIP-seq to improve the accuracy of identifying enhancers. Of particular interest is the incorporation of genome-wide assay for chromatin accessibility. Such experimental techniques, with examples including DNase-seq, MNase-seq, ATAC-seq, have being provided abundant data for not only studies of fundamental biological questions, but also applications to medical genetics and precision medicine. Second, our method can be used to determine deleterious SNPs in enhancers. Since our model can score the activity of an enhancer, it is natural to use our model to predict the impact of regulatory variants from sequence information.

Our model can certainly be improved in some aspects. First, convolutional neural networks are not suitable in dealing with sequences of variable length. Recent studies in recurrent neural networks have exhibited the success of the long short-term memory (LSTM) network, which is capable of handling sequential inputs of variable length and long-term dependencies. The incorporation of LSTM layers into our framework hence is natural and may produce even higher performance, since interactions of very long range in a sequence can be reasonably captured.

Second, our model can be extended to incorporate genomic information other than individual nucleotides. For example, we can alter the one-hot representation of A, C, G, T by adding information such as the multiple sequence alignment. From another perspective, we may also pre-train a vector representation of k-mers using unsupervised learning, such as GloVe [33], by investigating the co-occurrence matrix of k-mers, and use them to represent a DNA sequence. In this way, we can fuse the global genome information in representation of a local DNA sequence [34].

## Conclusions

We have proposed DeepEnhancer, a deep convolutional neural network framework, to distinguish enhancers from background sequences. Using FANTOM5 and ENCODE enhancer datasets with proper data preprocessing procedure, we trained several models with a variety of architectures and compared the classification performance with a traditional sequence-based method gkmSVM. We observed that our method surpassed the traditional approach in both effectiveness and efficiency. Besides, the use of max pooling and batch normalization can help improve the performance, while deeper models do not guarantee a better classification accuracy. Our model consistently outperformed gkmSVM for not only permissive enhancers but also enhancers specific individual cell lines, reflecting strong power of deep learning in capturing sophisticated features. To further promote the interpretability of our model, we transformed convolutional kernels in the first layer into position weighted matrices and then used a tool called TOMTOM to compare our PWMs against the JASPAR motif datasets. We found that our model can automatically learn meaningful motifs. Eventually, with the explosive growth of functional genomics data, we expect that such deep learning approaches will be broadly applicable and provide us highly accurate models.

## Methods

### Data sources

We collected two sets of enhancers from the FANTOM5 and ENCODE projects. Briefly, the FANTOM5 project systematically investigates how the genome encodes the diversity of cell lines that make up a human being. With an experimental technique called CAGE (cap analysis of gene expression), FANTOM maps transcripts, transcription factors, promoters and enhancers that are active in a majority of mammalian primary cell lines [9, 35]. The FANTOM project has published a package called promoter enhancer slider selector tool (PrESSTo) for users to select enhancers and promoters based on specific tissues and cell lines [36]. Using this tool, we obtain a total of 43,011 permissive enhancers. On the other hand, the ENCODE project provides tissue specific enhancers for 9 cell lines, including GM12878, H1-hESC, HepG2, HMEC, HSMM, HUVEC, K562, NHEK, and NHLF. We construct negative datasets by sample at random an equal number of background genome sequences. Here, we define the background genome as the entire human reference genome, excluding known enhancers, promoters for coding and noncoding genes, and exonic regions for coding and non-coding genes.

### Data augmentation

**Fig. 7:**
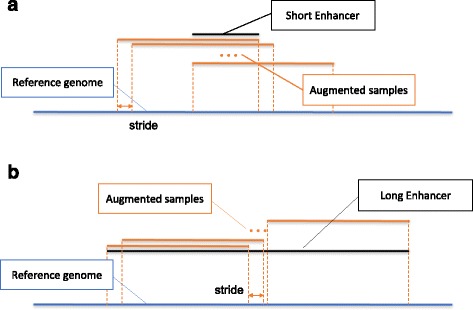
Diagram of data augmentation. Suppose the model accepts sequences of length *W* bps as input. **a** In the case that an enhancer is shorter than *W*, we slide a window of size *W* along the genome with stride *s* (default 2) around the input sequence, and take every sequence overlapping with the original one to obtain augmented sequences. **b** In the case that an enhancer is longer than *W*, we slide a window of size *W* along the input sequence with stride *s* (default 2) to obtain a number of sequences, each of length *W*

We consider two issues when implementing the deep neural network model. First, a convolutional layer only accepts sequences of fixed length as input, while enhancers in the FANTOM5 permissive dataset are of variable length. Second, a deep neural network requires a vast amount of training samples. We then propose a data augmentation strategy as illustrated in Fig. 7 to address both issues. Suppose sequences of length *W* (default to 300) are desired. In the case that an enhancer is shorter than *W*, we slid a window of size *W* along the genome with stride *s* (default 2) around the input sequence, and take every sequence overlapping with the original one to obtain augmented sequences. In the case that an enhancer is longer than *L*, we slide a window of size *W* along the input sequence with stride *s* (default 2) to obtain a number of sequences, each of length *W*.

With the above data augmentation strategy, we convert input sequences of variable length to short sequences of fixed length, at the same time greatly increased the number of available training sequences, i.e., completed the data augmentation procedure. We control the data augmentation ratio in a determinant way by changing the stride value. With the default value of 2, the number of permissive enhancers increases from 43,011 to about 1 million. In the training phrase, sequences augmented from enhancer regions are labeled as positive, and those from background regions are labeled as negative. In the test phase, we adopt a voting strategy to predict the probability that a sequence is an enhancer. Briefly, we use a trained model to score all sequences sampled from the original one, and we assign the maximum prediction probability to the original input sequence. The underlying principle is that we most care about whether part of the input sequence overlaps with a putative enhancer. If this is the case, there should exist some transcription factor binding sites (TFBS) or motif elements in the input sequence.

### Convolutional neural networks

Recent advances in computational biology have demonstrated successful applications of convolutional neural networks to the analysis of DNA sequences [37]. Typically, a convolutional layer, as the most crucial part in such a network, is composed of multiple convolutional kernels with equal size and is used to scan along the input DNA sequence for short patterns, in a manner analogous to a sliding window. A max-pooling layer, which often follows a convolutional layer, takes output of the preceded convolutional layer as input, and produces a maximal value as output. Such a pooling process is usually used to reduce the number of parameters to be learned and help to abstract features learned in the previous layers. An activation function is usually used after each layer to guarantee the nonlinearity of the whole model. A widely used activation function is the rectified linear unit (ReLU), defined as

$$ \mathrm{ReLU}(x)=\max \left(0,x\right). $$

In recent years, the batch-normalization layer has become popular [38], due to such benefit as the reduction of the internal covariate shift and the acceleration of the training procedure. On the top of the architecture are usually several fully connected layers, or dense layers, and a softmax layer playing the role of a nonlinear classifier based on the learned high level feature representation. The softmax function is a common used classifier in deep learning, which is a generalization of logistic regression classifier to multiple cases, as the following equation:

$$ {f}_i(z)=\frac{e^{z_i}}{\sum_j{e}^{z_j}}, $$

where *f*_*i*_(*z*) denotes the predicted score for class *i*. A dropout layer is used between fully connected layers, and it randomly sets input values to zero to avoid over-fitting [39]. The objective function to be optimized for a classification network is often the cross entropy loss, defined as the entropy between a true distribution *p* and the estimated class probabilities *q*, as

$$ \mathrm{H}\left(p,q\right)=-{\sum}_xp(x)\mathit{\log}q(x), $$

### Network architectures

We vary the architecture of the neutral network to investigate how different architectures affect the performance of a network. Seeking for the simplicity, we denote the default architecture in Table 1 as 4conv2pool4norm, which means the network has 4 convolutional layers, 2 max-pooling layers and 4 batch normalization layers. We use the same naming rule for the other architectures.

Dropping the batch-normalization layers, we obtain a variant architecture named 4conv2pool, for the purpose of exploring the effect of the batch normalization. We continue to throw away the max-pooling layers of 4conv2pool and obtain a variant architecture named 4conv for studying the influence of the max-pooling layers. To explore the impact of the network depth, we append 2 additional convolutional layers with 16 kernels of size 1 × 2 to make the CNN deeper, resulting another two variants 6conv3pool6norm and 6conv3pool. As such, we have a total of 5 different network architectures to be compared in our experiments.
